# Supplementary material for: COZOID: contact zone identifier for visual analysis of protein-protein interactions
Source: BMC Bioinformatics. 2018 Apr 6;19:125. doi: 10.1186/s12859-018-2113-6 (PMC5889581; doi:10.1186/s12859-018-2113-6)
Supplement: Supplementary file 2 — Software build. Executable binary file of the software tool. (ZIP 118,784 kb) [file 12859_2018_2113_MOESM2_ESM.zip › cozoid/platform/docs/testng-6.5.1-javadoc.zip!/org/testng/IHookCallBack.html]

IHookCallBack (TestNG)


---


|  |  |  |
| --- | --- | --- |
| **Overview** \| **Package** \| **Class** \| **Tree** \| **Deprecated** \| **Index** \| **Help** | |  |
| **PREV CLASS**   **NEXT CLASS** | **FRAMES**    **NO FRAMES**     **All Classes** |  |
| SUMMARY: NESTED \| FIELD \| CONSTR \| METHOD | DETAIL: FIELD \| CONSTR \| METHOD |  |


---


## org.testng Interface IHookCallBack

---

``` public interface IHookCallBack ```

A parameter of this type will be passed to the run() method of a IHookable.
Invoking runTestMethod() on that parameter will cause the test method currently
being diverted to be invoked.

**This interface is not meant to be implemented by clients, only by TestNG.**

**Author:**
:   cbeust
    Jan 28, 2006

**See Also:**: `IHookable`

---

| **Method Summary** | |
| --- | --- |
| `java.lang.Object[]` | `getParameters()` |
| `void` | `runTestMethod(ITestResult testResult)`             Invoke the test method currently being hijacked. |

**Method Detail**

### runTestMethod

```
void runTestMethod(ITestResult testResult)
```

:   Invoke the test method currently being hijacked.

---


### getParameters

```
java.lang.Object[] getParameters()
```

:   **Returns:**: the parameters that will be used to invoke the test method.


---


|  |  |  |
| --- | --- | --- |
| **Overview** \| **Package** \| **Class** \| **Tree** \| **Deprecated** \| **Index** \| **Help** | |  |
| **PREV CLASS**   **NEXT CLASS** | **FRAMES**    **NO FRAMES**     **All Classes** |  |
| SUMMARY: NESTED \| FIELD \| CONSTR \| METHOD | DETAIL: FIELD \| CONSTR \| METHOD |  |


---
